# Assessing rapid response mobile laboratory (RRML) capacities in Europe and Africa to improve One Health disease surveillance activities and pandemic preparedness across two continents

**DOI:** 10.1186/s42522-025-00180-6

**Published:** 2025-10-23

**Authors:** Anette Trojnacki, Julien A. Nguinkal, Jürgen May, Karin Rainer, Georg Gerhard Duscher, Lisa Winkelmayer, Ana Ungureanu, Eva Jaho, Angeliki Vlachostergiou, George Suciu, Cosmina Stalidi, Luminita Marcu, Holger Annus, Kristjan Kamdron, Andry Aasamäe, Martin Groschup, Klaas Dietze, Sascha Knauf, Markus Keller, Kassiani Mellou, Lida Politi, Georg Neubauer, Dražen Ignjatović, Aryse Martins Melo, Johannes R. Peham, Muna Affara, Florian Gehre

**Affiliations:** 1 Bernhard-Nocht-Institute for Tropical Medicine (BNITM), Hamburg, Germany; 2 German Center for Infection Research (DZIF), Partner Site Hamburg- Lübeck-Borstel-Riems, Hamburg, Germany; 3 Tropical Medicine II, University Medical Center Hamburg-Eppendorf (UKE), Hamburg, Germany; 4 Österreichische Agentur für Gesundheit und Ernährungssicherheit (AGES), Vienna, Austria; 5 Red Cross District 5, Bucharest, Romania; 6 EXUS AI Labs, Athens, Greece; 7 BEIA Consult International, Bucharest, Romania; 8 MDSC Systems OÜ, Tallinn, Estonia; 9 Friedrich-Loeffler-Institut (FLI), Federal Research Institute for Animal Health, Greifswald - Insel Riems, Germany; 10 Ethnikos Organismos Dimosias Ygeias, National Public Health Organization (NPHO), Athens, Greece; 11 Austrian Institute of Technology (AIT), Vienna, Austria

**Keywords:** Mobile laboratories, Pandemic preparedness, Emerging diseases, Disease outbreaks, Epidemics, Risk group 3, Risk group 4, BSL-4, Africa, Europe

## Abstract

**Background:**

Europe and Africa are increasingly affected by (re-)emerging risk group 3 and 4, zoonotic viral disease epidemics, which not only require diagnostic BSL-3/4 laboratory capacity but also a One Health-based control strategy for efficient outbreak containment. In many European and African countries such laboratory capacity is often not readily available, and rapid response mobile laboratories (RRMLs) can play important, complementary roles in outbreak responses and pandemic preparedness activities on national, regional and international level.

**Main body:**

The aim of the present review was to assess whether existing European and African RRML infrastructure is prepared for future One Health outbreak responses and to identify potential diagnostic gaps. Based on a literature review (2007–2021), we identified 291 mobile laboratories (Europe: 192, Africa: 99) and assessed them in respect to purpose (e.g. military, civilian), design (suitcase, modular, vehicle mounted), biosafety level, laboratory equipment, diagnostic portfolio, sample types analyzed (human, animal) and quality assurance measures. Following peaks in 2014 (Ebola/West Africa) and 2020 (COVID-19), mobile laboratory numbers have steadily increased. Whilst laboratories were originally designed to diagnose viral haemorrhagic fevers, there has been an increased focus on SARS-CoV-2 since 2020. Recently, there was a shift of African countries to develop an independent mobile laboratory capacity, rather than relying on external support for outbreak responses.

**Conclusion:**

We identified key shortcomings of existing laboratories, as the majority only process samples of human origin (not compliant with One Health principles), only 5% have sufficient capacity to diagnose emerging risk group 3/4 (arbo)viruses, 1–10% have accredited quality assurance systems in place, and mobile laboratories are not interconnected to allow concerted national and international responses. Our results reveal the gaps that should be addressed to make future responses to zoonotic, high-consequence pathogens more effective.

## Background

Africa is affected by numerous epidemics caused by pathogens of all risk groups, ranging from bacterial epidemics to viral haemorrhagic fevers caused by risk group 3 and 4 pathogens such as Ebola virus, Marburg virus, Lassa fever virus, dengue fever virus (DF), Mpox virus (MPXV), Crimean-Congo haemorrhagic fever virus (CCHFV) and West Nile virus (WNV) [1–5]. Due to climate change and expanding vector habitats, mosquito- and tick-borne arboviral diseases (WNV, DF, CCHFV) increasingly find their way from Africa into Europe. Southern Europe already saw autochthonous CCHFV outbreaks, and over the last twenty years became endemic for WNV epidemics. Although initially travel-related, DF is increasingly establishing itself in Europe as well [6]. As many of these (arbo)viral diseases have a zoonotic component in their transmission chain, a One Health approach for laboratory diagnosis and control is important for efficient outbreak containment.

While centralised laboratory infrastructure is available in some European and African countries, response times to new outbreaks are often long. Samples must be shipped from remote locations to national reference laboratories, and many remote regions still have limited laboratory capacity for risk group 3 and 4 pathogens. Rapid response mobile laboratories (RRML) can provide such specialised capacity and reduce the sample turnaround time at the site of the outbreak from days to same day results release for PCR-based assays [7]. This is important for individual patients (as they could be released from isolation wards/quarantine early), as well as for public health professionals to devise intervention strategies for containing epidemics as early as possible. Ultimately, RRMLs can bridge the diagnostic time gap, until the private sector, national agencies or international, emergency programs can establish the needed specialised, high-throughput diagnostic on-site capacity in regions affected by prolonged epidemics/pandemics.

In order to assess current RRML infrastructure in Europe and Africa, and whether it is suited for required One Health outbreak responses, we conducted a literature review and determined diagnostic gaps that need to be addressed in future RRML designs and projects.

## Literature review: search strategy and data evaluation

We conducted a literature research using PubMed, Google and Google Scholar. The search was restricted to the following keywords: “mobile laboratory” and the “name of the country” in Europe or Africa. All EU and EU’s associated countries [8], as well as all African countries were assessed. Although Tunisia is an EU-associated country it was assigned to Africa.

Selected articles and websites were abstracted based on following pre-defined, review-relevant criteria: civil or military use, laboratory structure, diagnostic equipment, biological safety level, quality management system, diagnosed pathogens, and the outbreak mission(s) including date. We stratified our laboratory analysis by continent (Europe vs. Africa) and diagnostic focus (“Conventional” diagnostics vs. “COVID-19”-specific laboratories). For laboratory design categorisation we followed the WHO classification for Rapid Response Mobile Laboratories (RRMLs), which discriminates between Type I (one to three highly mobile, compact units, e.g. laboratory in suitcases); Type II (more than three modular, box-based mobile laboratory units); Type III are self-contained, medium units in smaller vehicles, such as pick-ups; Type IV are large scale, individual truck-mounted container-based laboratories; Type V laboratories are fleets of full-scale Type IV laboratories or Type IV laboratories with significant external structures [9].

Literature describing biological or medical uses of a mobile laboratory was appraised and included if at least one of the review-relevant criteria was described. For literature, which only partially contained the pre-defined criteria, the respective missing information was classified as “not available”. The date and time of assessment of articles, websites and newspapers was documented. Access of articles were documented by month and websites with the exact date and time. The first reference was accessed on 28 July 2021 at 14:59 and last on 29 November 2021 at 17:01. Secondary references in the selected literature were also viewed and included in the data of this review, as well as non-peer-reviewed news articles and websites.

In total 821 articles were identified and 774 of those were used for the evaluation. 47 articles were excluded because they did not provide sufficient information on predefined criteria (as described above), the owner or country was not specified, or the laboratories were not used in Europe or Africa. Mobile laboratories with no biological or medical context were also excluded.

### Mobile laboratory capacity in Europe and Africa (2007–2021)

**Fig. 1:**
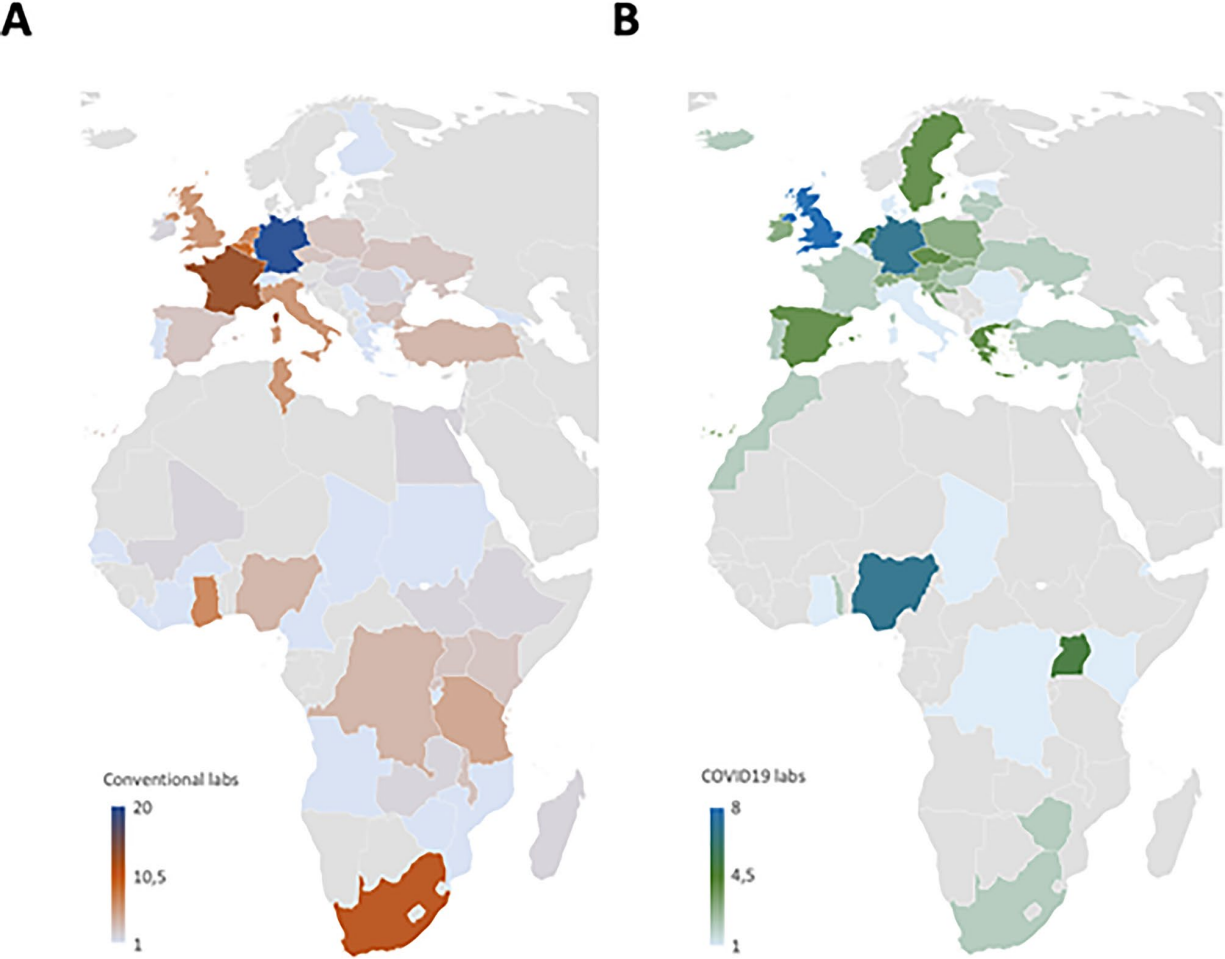
Heatmap showing the presence of mobile laboratories in Europe and Africa. **(A)** Number of conventional mobile laboratories, **(B)** Number of laboratories specifically designed for SARS-CoV-2 diagnostics (COVID-19 laboratories)

A total number of 192 mobile laboratories in Europe and 99 mobile laboratories in Africa were identified. 39 out of 43 European countries and 32 out of 54 African countries had mobile laboratories (Fig. 1; Table 1).

**Table 1:** Amount of mobile laboratories and respective diagnostic portfolios by country

Country	Number of laboratories	Diseases diagnosed
**Europe**		
Armenia	1	COVID-19
Austria	3	COVID-19
Belgium	10	Anthrax, chikungunya virus, COVID-19, dengue virus, Ebola virus, flavivirus, human metapneumovirus, influenza, Malaria, Mpox virus, Orthopoxvirus, *Plasmodium* spp., rash illness, respiratory syncytial virus, Rift Valley Fever, *Salmonella* spp., Smallpox, varicella virus
Bulgaria	4	HIV, COVID-19
Croatia	3	COVID-19
Cyprus	2	COVID-19
Czech Republic	7	COVID-19, Ebola virus, anthrax
Denmark	1	COVID-19
Estonia	1	COVID-19
Faroe Island	1	COVID-19
Finland	1	
France	15	blood stream infection, COVID-19, dengue virus, Ebola, HIV, Malaria, meningitis
Georgia	3	COVID-19
Germany	25	avian influenza virus, COVID-19, Ebola virus, *H. ducreyi*, HIV, influenza A virus subtype H1N1 and H7N9, *L. donovani*, Malaria, Marburg virus, *T. pallidum*, tuberculosis, viral haemorrhagic fevers, visceral leishmaniose, yaws, yellow fever virus
Greece	5	COVID-19
Hungary	4	COVID-19,
Iceland	2	COVID-19
Ireland	5	COVID-19, microbiological testing
Italy	7	COVID-19, Ebola virus, HIV, Malaria
Israel	4	COVID-19, Ebola virus
Latvia	2	COVID-19
Lithuania	2	COVID-19
Luxembourg	4	COVID-19, Ebola virus
Malta	2	Hepatitis
Moldova	1	*B. anthracis*, COVID-19
Netherlands	12	COVID-19, Ebola virus, Malaria, Zika virus
North Mazedonia	2	COVID-19
Poland	6	avian influenza virus, avian influenza A virus, COVID-19, *E. coli*, influenza B virus, parainfluenza type 1, 2, 3, *V. cholera*, *Y. pestis*
Portugal	3	COVID-19, Ebola virus
Romania	3	COVID-19
Serbia	1	
Slovakia	5	COVID-19
Slovenia	5	COVID-19
Spain	7	COVID-19
Sweden	4	chikungunya virus, COVID-19, HIV, *L. brasiliensis*, *L. donovani*, Malaria, tuberculosis, visceral leishmaniose
Switzerland	4	COVID-19
Turkey	6	COVID-19
United Kingdom	14	Capripoxvirus, COVID-19, Ebola, Malaria
Ukraine	5	COVID-19, HIV
**Africa**		
Angola	1	
Burkina Faso	1	Meningitis, *V. cholera*
Burundi	1	COVID-19, Ebola virus
Cameroon	1	
Chad	2	COVID-19
Cote d’lvoire	1	sleeping sickness
Democratic Republic of Congo	5	COVID-19, Ebola virus, sleeping sickness
Djibouti	1	COVID-19
Egypt	2	Influenza virus, Rift Valley fever virus
Ethiopia	2	Emerging virus, viral haemorrhagic fevers
Gambia	1	Meningitis
Ghana	8	COVID-19, Malaria
Kenya	4	COVID-19, Ebola virus
Liberia	1	Ebola virus
Madagascar	2	Enteropathogens, COVID-19, Ebola virus, Malaria, measles, plague, protozoa,
Malawi	3	Malaria
Mali	2	COVID-19, Ebola virus, Lassa virus, Rift Valley fever virus, Zika virus
Morocco	2	COVID-19
Mozambique	1	HIV
Nigeria	11	COVID-19, Ebola virus, meningitis
Rwanda	3	COVID-19, Ebola virus, Hepatitis C
Senegal	1	COVID-19, Crimean-Congo haemorrhagic fever virus, dengue virus, Ebola virus, Marburg virus, Rift Valley fever virus, yellow fever virus
Seychelles	1	
South Africa	13	COVID-19, Ebola virus, Hepatitis A, Hepatitis E, HIV, HPV, tuberculosis, Zika virus
South Sudan	2	COVID-19, Ebola virus
Sudan	1	
Tanzania	5	COVID-19, Ebola virus, tuberculosis
Togo	2	COVID-19
Tunisia	6	COVID-19, infant diarrheal disease, Malaria
Uganda	8	COVID-19, Ebola virus
Zambia	2	Contagious bovine pleuropneumonia, HIV, tuberculosis
Zimbabwe	3	COVID-19, HIV

**Fig. 2:**
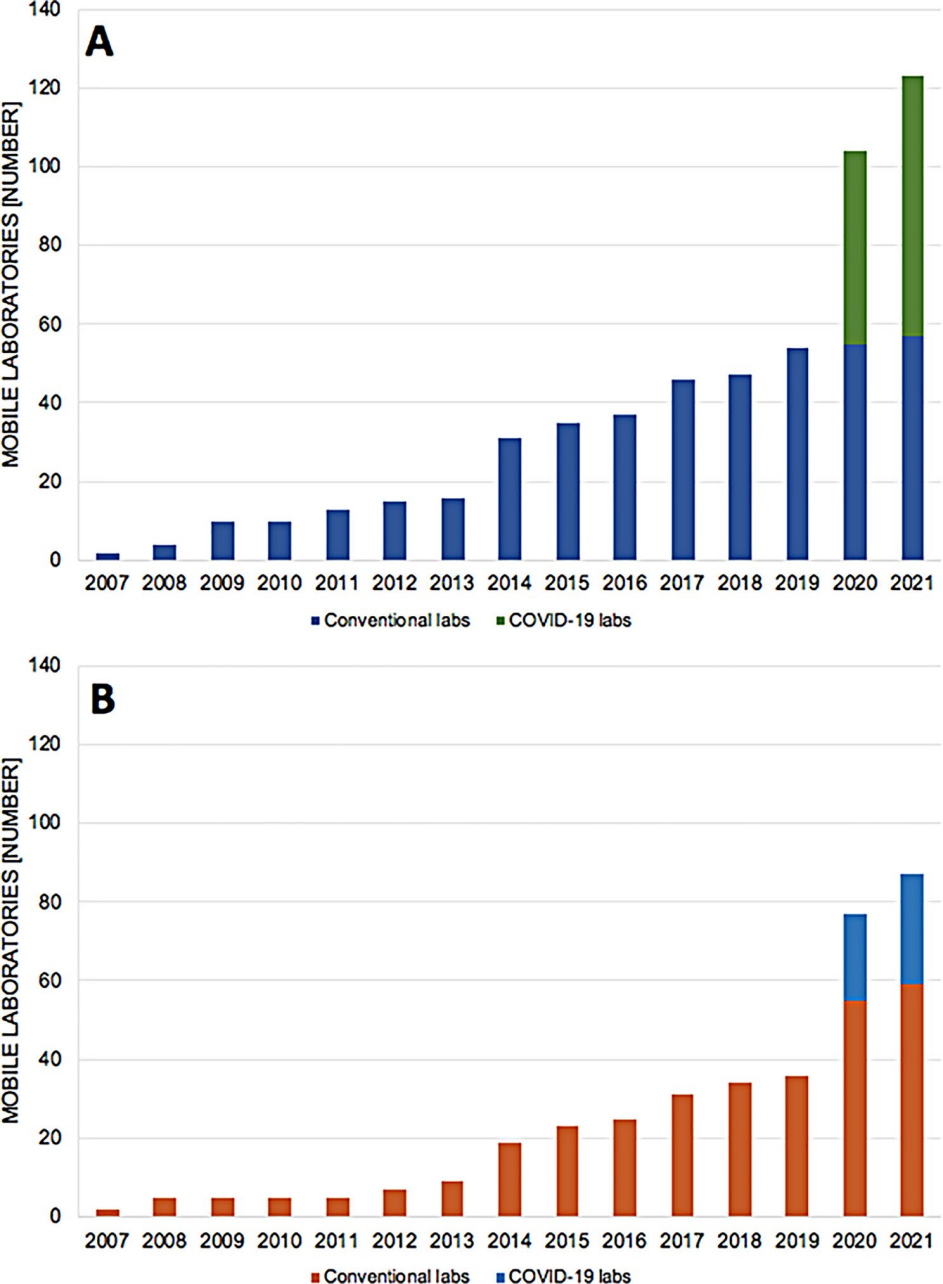
Cumulative development of conventional and COVID-19 laboratory between 2007 and 2021. **(A)** in Europe, **(B)** in Africa. It is of note that not all mobile laboratories mentioned an inception date, and are therefore not captured in the figure

Germany, UK, France, Nigeria, Uganda and South Africa had the most laboratory initiatives. Although not all reports stated a laboratory project inception date, we observed increasing mobile laboratory numbers between 2007 and 2021 (Fig. 2).

Following 2014, a first peak of mobile laboratory numbers was in response to the West African Ebola epidemic, and in 2021 a second peak was attributable to specialized SARS-CoV-2 laboratories during the COVID-19 pandemic.

### Mobile laboratory design, purpose and biosafety levels

**Fig. 3:**
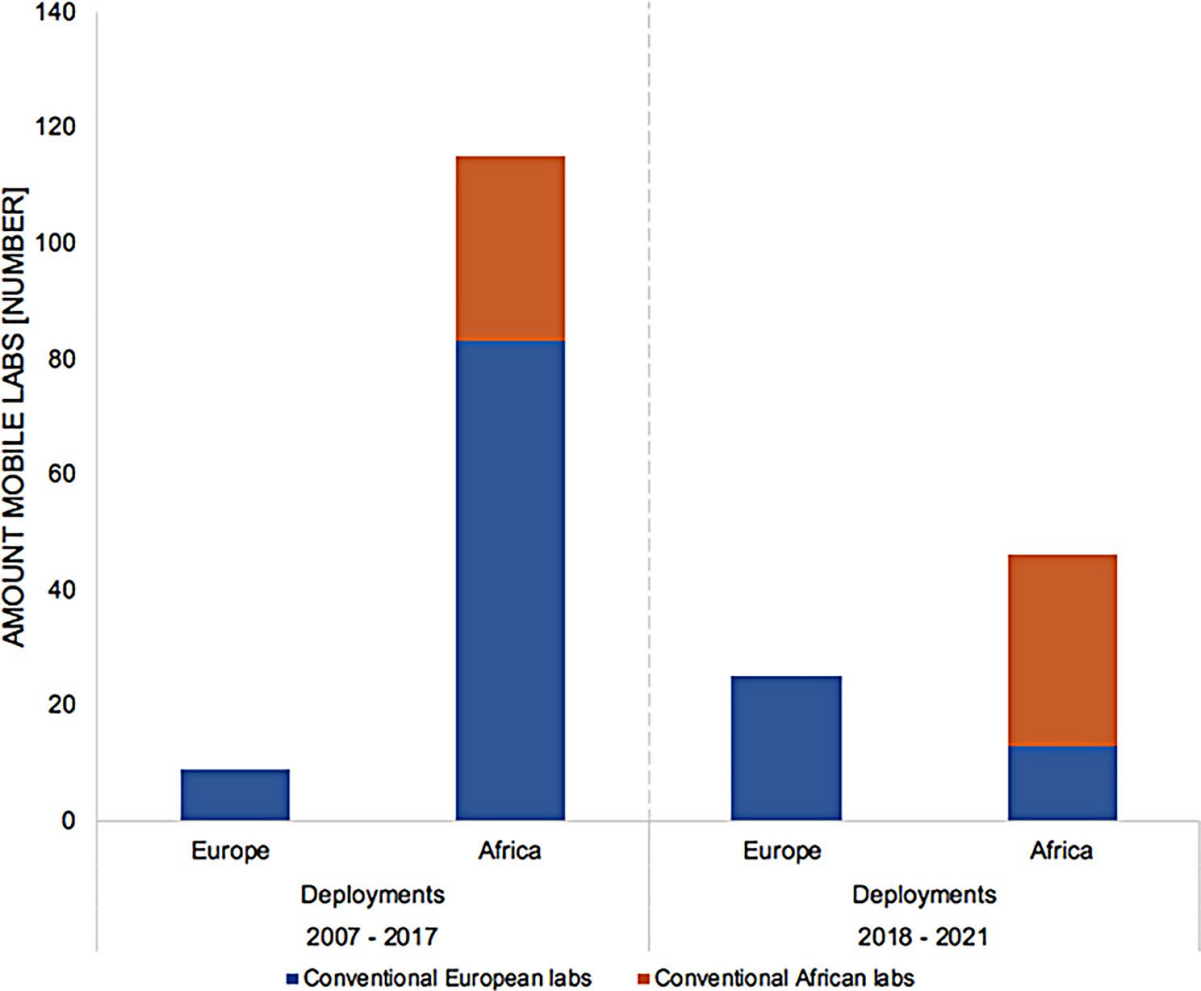
Deployment destinations of European and African conventional laboratories. Between 2007 and 2017 the majority of mobile laboratory missions in Africa were achieved by conventional mobile laboratories originating from Europe. Following 2018, a shift was observed, and the majority of mobile laboratory missions in Africa were conducted by mobile laboratory projects from Africa, whereas European mobile laboratories focussed on laboratory activities within Europe

We observed that the majority of laboratories were for civilian use, yet in Europe a higher proportion of conventional mobile laboratories was operated by the military (19%) or with dual civilian/military (15%) purpose (Table 2), probably due to the fact that many mobile laboratory concepts were originally designed by the military [10, 11] and later handed over to civilian partners in Europe and Africa. Most laboratories in Africa were type III, IV and V. In Europe, a higher proportion of laboratories utilised a type I or II design when compared to Africa (31% and 21%, respectively) (Table 2). This more portable design preference was due to the fact that European mobile laboratory initiatives between 2007 and 2017 were often designed to be airfreighted to assist in African outbreaks. Between 2007 and 2017 over 60% of field missions in Africa were still conducted by European laboratories (Fig. 3).

**Table 2:** Mobile laboratory characteristics in Europe and Africa

	European Mobile Laboratories (*n* = 192)				African Mobile Laboratories (*n* = 99)			
	Conventional Laboratories (*n* = 101)		COVID-19 Laboratories (*n* = 91)		Conventional Laboratories (*n* = 69)		COVID-19 Laboratories (*n* = 30)	
	n	%	n	%	n	%	n	%
**Laboratory purpose**								
Civilian	58	57%	72	79%	62	90%	24	80%
civilian/military	15	15%	3	3%	1	1%	1	3%
Military	19	19%	2	2%	5	7%	-	-
no information	9	9%	14	15%	1	1%	5	17%
**Laboratory Design (according to WHO classification for RRMLs)**								
Highly compact suitcase lab (Type I)	14	13%	5	5%	2	2%	-	-
Box - based /modular lab (Type II)	12	11%	2	2%	13	16%	1	3%
Medium to Large scale self-contained laboratories in mobile vehicles (Type III-Type V)	56	50%	49	53%	26	32%	11	37%
no information	29	26%	36	39%	40	49%	18	60%
**Biosafety Level**								
Risk group 2 pathogens	7	7%	10	11%	2	3%	-	-
Risk group 3 pathogens	21	21%	5	5%	5	7%	-	-
Risk group 4 pathogens	5	5%	-	-	9	13%	2	7%
no information	68	67%	76	84%	53	77%	28	93%
**Sample types**								
Human	73	72%	91	100%	61	88%	30	100%
non-human	2	2%	-	-	2	3%	-	-
Both	19	19%	-	-	3	4%	-	-
no data	7	7%	-	-	3	4%	-	-
**Diagnostic Equipment**								
PCR	32	26%	56	43%	25	27%	11	35%
ELISA	8	6%	3	2%	13	14%	-	-
RPA (= recombinase polymerase assay)	4	3%	-	-	1	1%	-	-
IFA/ ICT (= immunochromatographic assay)	5	4%	1	1%	-		-	-
Serological tests	1	1%	5	4%	1	1%	-	-
Unknown	57	46%	25	19%	37	41%	18	58%
Other diagnostics	17	14%	41	31%	14	15%	2	6%
**Diseases diagnosed during missions**								
Arbovirus and other haemorrhagic fever viruses	113	43%	-	-	38	48%	-	-
Covid-19	20	8%	91	100%	16	20%	49	98%
HIV/ AIDS	5	2%	-	-	2	3%	-	-
Influenza	10	4%	-	-	-		-	-
other virus	27	10%	-	-	2	3%	-	-
Malaria and other parasites	34	13%	-	-	1	1%	-	-
Bacterial Disease	17	6%	-	-	9	11%	-	-
Unspecific	11	4%	-	-	1	1%	-	-
Other	27	10%	-	-	11	14%	1	2%
**Quality Assurance**								
QMS – accredited	9	8%	10	10%	1	1%	-	-
QMS – LIMS	4	4%	2	2%	2	3%	-	-
QMS – other	31	28%	43	43%	26	36%	10	33%
no QMS	67	60%	45	45%	43	60%	20	67%

Following 2017, we observed a policy shift with the establishment of independent mobile laboratories in Africa, and between 2018 and 2021 (Fig. 2) more than 80% of field missions were conducted by African laboratories (Figs. 2 and 3).

7% of African laboratories have the capacity to handle risk group 3 pathogens and 13% risk group 4 pathogens. In Europe 21% can diagnose risk group 3 pathogens and only 5% risk group 4 pathogens (Table 2).

### Mobile laboratory diagnostic portfolios and one health capacity

The majority of mobile laboratories in Africa and Europe were designed for exclusive processing of human samples. 2–3% of laboratories exclusively process animal samples, and 19% of conventional European and 4% of conventional African laboratories have the capacity to process samples of human and animal origin (according to a One Health mandate). For an overview of diagnosed disease per country, see Table 1.

The most frequent diagnostic tool in mobile laboratories are PCR-based technologies, followed by serology tests such as ELISA (Table 2). The predominance of molecular PCR-based diagnostics is a reflection of the purpose of mobile laboratories: the majority of laboratories were designed to diagnose viral outbreaks, where RT-PCR is the diagnostic of choice. Generally, PCR-based results can be generated within the same day of sample reception. In addition, container-based mobile laboratories were utilised for bacterial diagnostics on both continents (Table 2).

### Quality assurance in existing mobile laboratories

We assessed whether existing mobile laboratory initiatives established any quality standards or quality management systems (QMS) for disease diagnostics. We only found around 8% − 10% of all European and around 1% of all African mobile laboratories were documented/reported as accredited by independent international organizations. The majority (> 50%) of mobile laboratories did not document having a QMS in place.

## Conclusions

We conducted an inventory of all mobile laboratory infrastructure established in Europe and Africa. The importance of mobile laboratories in providing specialised diagnostic capacity to remote or cross-border areas and therefore reducing diagnostic sample turn-around-times was demonstrated during two epidemic key events: during the Ebola epidemic in West Africa in 2014 [11–19], and the COVID-19 pandemic which started in 2020. Because of their increasing importance in outbreak responses, WHO Europe was consequently developing minimum operational standards for RRMLs [7].

Our inventory, however, revealed several shortcomings of existing mobile laboratories in particular for their use in future outbreak responses to (emerging) zoonotic pathogens, which could inform the design of the next mobile laboratory generation:

1. **Lack of “One Health” aspect**: almost all (88%) existing mobile laboratory diagnostics exclusively focus on human patients. It is increasingly appreciated that neglecting the animal domain of zoonotic pathogen’s life cycle will negatively impact on any outbreak response and maintain chains-of-transmission. Although we do not want to assert that all mobile laboratories need this capacity, the incorporation of One Health sample workflows into novel mobile laboratories, especially those designed to respond to emerging and re-emerging vector-borne infections would benefit existing arboviral control programmes.
2. **Lack of Quality Control Systems**: only 7% (20/291) of existing mobile laboratories have an accredited Quality Management System.
3. **Lack of risk group 4 pathogen handling capacity**: Considering that even in most European and African countries stationary BSL-4 laboratories capacity is still a scarcity, one of the biggest impacts that mobile laboratories can have, is the provision of temporary, decentralised diagnostic capacity for risk group 4 pathogens. Although not needed for all mobile laboratories, increasing the proportion of mobile laboratories with glove boxes will be very beneficial for pandemic preparedness activities of many European and African countries.
4. **Lack of interoperability and internationalisation of existing mobile laboratories to tackle cross-border pandemics**. Existing European and African mobile laboratory initiatives are not interconnected and therefore orchestrated regional outbreak responses are difficult to coordinate.

### Limitations of the study

Our study also has technical limitations. We were not able to include data of mobile laboratories that were currently in the field, for which no publication or grey literature existed at the time of our literature research. Similarly, military infrastructures might be underreported. Moreover, as the landscape of mobile diagnostics (e.g. novel Point-of-care tests, telemedicine, mobile sequencing) is at the moment rapidly changing, the review might have missed certain key developments in the field of mobile laboratories.

Our review, however, demonstrates the increasing importance of mobile laboratories to epidemic and pandemic preparedness in Europe and Africa, yet also identified current design shortcomings. Knowledge of the availability of all mobile laboratory resources in Europe and Africa will enable first responders, National Agencies, Ministries of Health, National Public Health and Veterinary Laboratories and other international stakeholders. This is especially important to efficiently tackle cross border epidemics and intercontinental pandemics.

## Data Availability

The datasets used and/or analyzed during the current study are available from the corresponding author on reasonable request.

## Abbreviations

DF: Dengue Fever
MPXV: Mpox virus
CCHFV: Crimean-Congo haemorrhagic fever virus
WNV: West Nile virus
RRML: Rapid response mobile laboraotry
RT-PCR: Real time polymerase-chain reaction
ELISA: Enzyme-linked immunosorbent assay
QMS: Quality management system
BSL: Biosafety level
RPA: Recombinase polymerase assay
IFA: Immunofluorescence assays
ICT: Immunochromatogrphic assay
LIMS: Laboratory Information management system
